# Editorial: Interpreting the Comorbidity of Learning Disorders

**DOI:** 10.3389/fnhum.2021.811101

**Published:** 2021-12-09

**Authors:** Kristina Moll, Maria De Luca, Karin Landerl, Chiara Banfi, Pierluigi Zoccolotti

**Affiliations:** ^1^Department of Child and Adolescent Psychiatry, Psychosomatics, and Psychotherapy, Ludwig-Maximilians University, Munich, Germany; ^2^Developmental Dyslexia Lab, IRCCS Fondazione Santa Lucia, Rome, Italy; ^3^Institute of Psychology, University of Graz, Graz, Austria; ^4^BioTechMed-Graz, Graz, Austria; ^5^School of Psychology, Macquarie University, Sydney, NSW, Australia; ^6^Institute for Medical Informatics, Statistics and Documentation, Medical University of Graz, Graz, Austria; ^7^Department of Psychology, Sapienza University of Rome, Rome, Italy

**Keywords:** comorbidity, dyslexia, dyscalculia, ADHD, motor disorders, learning

Reading, spelling, and arithmetic are crucial domains of school achievement, and neurodevelopmental learning disorders in written language processing (dyslexia) and arithmetic (dyscalculia) have a marked impact on children's academic careers and professional perspectives (Ritchie and Bates, 2013). Prevalence studies clearly show high rates of co-occurrence (comorbidity) between these learning disorders as well as with other neurodevelopmental disorders, such as attention-deficit-hyperactivity disorder (ADHD), developmental language disorder, or even developmental motor disorder, so that the concept of “*specific*” learning disorders, affecting one learning domain only, is seriously challenged.

The high co-occurrence rate between learning disorders has also been acknowledged in the latest edition of the Diagnostic and Statistical Manual of Mental Disorders (DSM-5; American Psychiatric Association, 2013) which now places a variety of disorders across several learning domains (i.e., reading decoding and comprehension, spelling and written expression, number sense, and mathematical reasoning) under a single diagnostic category. Still, the category used (“*Specific Learning Disorder*”) maintains the ambiguous term “specific” presumably with the aim to highlight that deficits in learning are not due to other developmental disorders or intellectual disabilities.

## The Role of Comorbidity for Advancing Causal Models of Learning Disorders

In the past, our knowledge on the manifestation and causation of neurodevelopmental learning disorders has derived mostly from studies investigating each learning disorder separately (either dyslexia or dyscalculia). These studies have often either deliberately excluded individuals with additional learning problems, interpreted such problems as a consequence of the disorder studied, or simply neglected co-occurring disorders. However, single deficit models may not provide good explanations for the high *heterogeneity* in the symptomatology of learning disorders. Overall, there is growing consensus that the etiology of neurodevelopmental disorders is best interpreted within a *multiple-deficit framework* (Pennington, 2006; McGrath et al., 2020). This aims to provide the theoretical background for explaining both co-occurrence of and dissociations between, disorders. Comorbidity is explained by risk factors that are shared between disorders, while dissociations are explained by disorder-specific risk factors. The pattern of symptoms in individual cases is also likely to be influenced by protective factors, which however are rarely considered in research. Altogether multiple risk and protective factors determine the behavioral outcome (Pennington et al., 2012) and may thus provide a better account for the heterogeneity observed in neurodevelopmental disorders.

Risk (and protective) factors are probabilistic and interact with each other, with some factors being more relevant and more specific for a certain disorder than others. The search for these factors represents a new and challenging field of research. Several relevant cognitive risk factors have been identified that influence a child's susceptibility to developing a single disorder (e.g., a phonological deficit in dyslexia) or a combination of learning disorders (e.g., deficits in language, working memory, or executive functions). Still, the complex interplay between these risk factors and thus the mechanisms underlying the large variability of individual profiles are not well-understood.

Similarly, neurobiological studies have identified differences in brain structure and function associated with a single disorder as well as differences potentially associated with the overlap between learning disorders. For example, much research has aimed to uncover structural and functional differences both in the case of dyslexia (e.g., Richlan et al., 2009; Ozernov-Palchik et al., 2016) and dyscalculia (Landerl et al., 2021; Vogel and De Smedt, 2021). However, studies explicitly investigating the neuronal overlap between dyslexia and dyscalculia and the complex interplay between the different levels of analyses (i.e., neurobiological and cognitive) and the behavioral manifestations are as yet rare (see for example Peters et al., 2018). Similar considerations may apply to the overlap between learning disorders and other developmental disorders, such as ADHD and motor disorders (Pennington et al., 2019).

The present Research Topic (RT) brings together a number of studies that try to elucidate cognitive risk (and protective) factors focussing particularly on the relationship between reading and math skills (and deficits) but also considering other disorders such as ADHD and motor difficulties, as well as protective factors (such as cognitive strengths), helping children to compensate for their learning disorders.

## Cognitive Factors Underlying the Relationship Between Reading and Math Skills

Dyslexia is characterized by significant and persistent difficulties related to reading, such as reading accuracy, fluency, or comprehension. About 40% of children with reading problems also have low spelling skills (Moll and Landerl, 2009; Moll et al., 2014). Dyscalculia also has a broad range of manifestations, including deficits in numerical abilities, i.e., understanding and processing of non-symbolic numerosity and/or its symbolic representations (Arabic numbers and number words), deficits in arithmetic, i.e., mental calculations, fact retrieval and calculation procedures, and problems in math reasoning. Prevalence studies consistently report high comorbidity rates between dyslexia and dyscalculia, ranging between 11 and 70% (for an overview see: Moll et al., 2014), depending on cut-off criteria applied and tasks and constructs (and thus symptoms) used to define the disorders (Dirks et al., 2008; Landerl and Moll, 2010).

Various studies in the RT jointly examined reading and math skills in samples of typically developing children with the aim to elucidate the cognitive factors which may account for the overlap among these skills (and potentially increase the risk of developing both a reading and math problem). Bernabini et al. used a dimensional approach to examine the relationship between reading and math in a sample of 4^th^- and 5^th^-grade children. Their approach envisaged both examining the influence of reading and math skills on their putative cognitive predictors as well as the opposite, that is the influence of cognitive abilities in predicting reading and math. These two ways of looking at data provide interesting complementary information on the overlap between reading and math skills. In a carefully planned longitudinal study, Amland et al. examined whether the quality of phonological representations could provide the possible foundation of the association between reading and math skills (as well as disorders). Results did not show a direct effect of phonological awareness in arithmetic development, although an indirect influence of this parameter did emerge on verbal arithmetic (but not fluency). The authors emphasize the importance of accurate control of all possible confounders in the examination of common risk factors. Geary et al. examined the contribution of general cognitive abilities (including intelligence, verbal short-term and working memory, visuospatial memory, attention, and ability measures) and academic attitudes (particularly in-class attentive behavior) in predicting reading and math achievement (separately as well in co-morbidity fashion).

A complementary (though less frequently investigated) perspective is that of examining cognitive strengths that may help children to compensate for their learning disorders. Huijsmans et al. carried out one such study including children with isolated mathematical learning difficulties and children with comorbid mathematical and reading difficulties. Data indicated that strong rapid naming skills provided partially effective mechanism for children with math deficiencies (though not for children with both reading and math problems).

In understanding the overlap between reading and math difficulties, important aspects to consider are the learning environment at home as well as the presence and type of parental difficulties on the development of reading and math skills. Khanolainen et al. carried out a large longitudinal study examining these factors. The results indicated the interrelated role of familial risk, parental education, and type of learning environment at home in shaping the acquisition of math and reading skills.

Examining disorders from a comorbidity perspective may also help in pinpointing a more comprehensive description of the disorder. Kißler et al. investigated the possible presence of subtypes of dyscalculia in two samples in which the diagnosis was based either with a focus on calculation or on numerical capacities. Independent of the type of diagnosis results based on a mixture model analysis revealed the presence of two main sub-types of dyscalculia. The main difference was in terms of the degree of math impairment but also differences in attention skills contributed to the distinction, indicating the role of comorbidity in shaping dyscalculia subtypes.

## Modeling Reading, Spelling, and Math

The growing knowledge about the common factors underlying reading, spelling and math creates the necessary premise to build a unitary architecture of these skills (and deficits).

Based on data from a group of typically developing children, Zoccolotti et al. proposed a multi-level model to account for the association among reading, spelling, and math skills which capitalize on the distinction among competence, performance, and acquisition (automatization). The model aims to provide a heuristic to account for the comorbidity of learning disorders in these areas, in particular with the aim of explaining both dissociations (related to the presence of distinct competencies) and associations (related to the influence of common performance factors as well as to the widespread effect of deficits in automatization).

## Specific Learning Disorders and ADHD

A well-known association with learning disorders concerns the ADHD symptomatology (e.g., Pham and Riviere, 2015). Jointly examining individuals with both dissociated and associated symptomatology may prove as an effective paradigm for the understanding of both disorders.

In a study using latent profile analysis, Laasonen et al. examined how measures based on different non-verbal theories (including temporal processing impairment, abnormal cerebellar functioning, procedural learning difficulties, visual processing, and attention deficits) would allow classifying adults with dyslexia, ADHD, or both. The authors showed that participants did not cluster according to their original diagnosis and thus underscored the “*continuous and overlapping nature of the observed difficulties*.”

Crisci et al. examined the possible role of comorbidity between specific learning disorders (SLD) and ADHD on executive functions by testing children with either SLD, ADHD, or both. Results indicated a widespread association of SLD, ADHD with inhibition and shifting tasks as well as a more selective influence on updating tasks. While children with SLD were impaired in verbal updating those with ADHD or with both SLD and ADHD were most impaired in spatial updating. Thus, it appears that considering the comorbidity between SLD and ADHD is important for a better understanding of both disorders.

## Literacy and Motor Disorders

Some evidence indicates that comorbidity encompasses a wide spectrum of developmental disorders including both cognitive and motor difficulties (e.g., Cruddace and Riddell, 2006), though the nature of this comorbidity is still poorly understood.

Downing and Caravolas examined the possible association between reading and motor difficulties and evidenced a high co-morbidity between the two; indeed, the joint presence of literacy and motor difficulties was five times higher than what was expected based on the prevalence rates for each disorder (Study 1). In a further study, they searched for both independent and shared factors in the cognitive profile of these disorders: phonological processing and selective attention were risk factors for literacy disorders and visuospatial processing for motor disorders. Memory proved as a risk factor for the comorbid presence of literacy and motor disorders. These results confirm that also motor disorders can be interpreted within a multi-factorial perspective (Pennington, 2006).

## Conclusion

The working hypothesis guiding the present RT was that single deficit models do not provide good explanations for the high *heterogeneity* in the symptomatology of learning disorders. In keeping with this idea, various studies in this RT provide new information on the characteristics of several developmental disorders (including dyslexia, ADHD, motor difficulties). Additional information in this RT comes from studies of typically developing children which also provide clues as to factors that underly the co-variation among reading, spelling, and math skills. It appears that to see skills (and relative deficits) in a comorbidity perspective represents an effective prospect to understand a wide spectrum of developmental disorders.

## Author Contributions

KM and PZ: wrote part of the first draft of the paper. MD, KL, and CB: made several changes to various versions of the manuscript. All authors gave a similar contribution.

## Funding

This work was partially supported by the Italian Ministry of Health (Ministero della Salute, Ricerca Corrente) to IRCCS Fondazione Santa Lucia (Research Line 4 – Cognitive and Motor Neurorehabilitation and Neuroimaging).

## Conflict of Interest

The authors declare that the research was conducted in the absence of any commercial or financial relationships that could be construed as a potential conflict of interest.

## Publisher's Note

All claims expressed in this article are solely those of the authors and do not necessarily represent those of their affiliated organizations, or those of the publisher, the editors and the reviewers. Any product that may be evaluated in this article, or claim that may be made by its manufacturer, is not guaranteed or endorsed by the publisher.
